# *Saccharomyces cerevisiae* transcriptional reprograming due to bacterial contamination during industrial scale bioethanol production

**DOI:** 10.1186/s12934-015-0196-6

**Published:** 2015-01-30

**Authors:** Osmar V Carvalho-Netto, Marcelo F Carazzolle, Luciana S Mofatto, Paulo JPL Teixeira, Melline F Noronha, Luige AL Calderón, Piotr A Mieczkowski, Juan Lucas Argueso, Gonçalo AG Pereira

**Affiliations:** Departamento de Genética, Evolução e Bioagentes, Instituto de Biologia, Universidade Estadual de Campinas, Campinas, SP Brazil; Department of Environmental and Radiological Health Sciences, Colorado State University, Fort Collins, CO USA; Department of Genetics, University of North Carolina, Chapel Hill, NC USA

**Keywords:** *Saccharomyces cerevisiae*, Bioethanol, Co-aggregation, Transcriptome, RNA-seq

## Abstract

**Background:**

The bioethanol production system used in Brazil is based on the fermentation of sucrose from sugarcane feedstock by highly adapted strains of the yeast *Saccharomyces cerevisiae*. Bacterial contaminants present in the distillery environment often produce yeast-bacteria cellular co-aggregation particles that resemble yeast-yeast cell adhesion (flocculation). The formation of such particles is undesirable because it slows the fermentation kinetics and reduces the overall bioethanol yield.

**Results:**

In this study, we investigated the molecular physiology of one of the main *S. cerevisiae* strains used in Brazilian bioethanol production, PE-2, under two contrasting conditions: typical fermentation, when most yeast cells are in suspension, and co-aggregated fermentation. The transcriptional profile of PE-2 was assessed by RNA-seq during industrial scale fed-batch fermentation. Comparative analysis between the two conditions revealed transcriptional profiles that were differentiated primarily by a deep gene repression in the co-aggregated samples. The data also indicated that *Lactobacillus fermentum* was likely the main bacterial species responsible for cellular co-aggregation and for the high levels of organic acids detected in the samples.

**Conclusions:**

Here, we report the high-resolution gene expression profiling of strain PE-2 during industrial-scale fermentations and the transcriptional reprograming observed under co-aggregation conditions. This dataset constitutes an important resource that can provide support for further development of this key yeast biocatalyst.

**Electronic supplementary material:**

The online version of this article (doi:10.1186/s12934-015-0196-6) contains supplementary material, which is available to authorized users.

## Background

Brazilian bioethanol is mainly produced from the fermentation of sugarcane juice and molasses by *Saccharomyces cerevisiae*. Yeast cells are added at the beginning of the production season and are recycled at the end of each fed-batch fermentation cycle, every 8 to 15 hours, for approximately 210 consecutive days. Because the feedstock is not completely sterilized prior to fermentation, microbial contaminants are continuously introduced to the distillery environment, resulting in a dynamic competition between the desired inoculated strain and wild yeast strains and bacteria [1-3].

PE-2 and CAT-1 are the most versatile and widely adopted *S. cerevisiae* strains used by Brazilian distilleries [1]. Previously, we described the genome structure of the JAY270 strain, a clonal isolate derived from a commercial PE-2 stock [4]. That study provided initial insights into the genetic mechanisms that underlie the strong performance of this strain as an industrial biocatalyst. JAY270 is a heterothallic diploid strain, and its genome is characterized by a high degree of heterozygosity. This intrinsic genetic diversity is likely a key factor in the extraordinary ability of PE-2 to thrive in the harsh environment found in industrial fermentation tanks. PE-2 typically persists for the whole production season as the dominant strain in the yeast population, stemming the proliferation of wild contaminant yeast strains.

Yeast flocculation is a phenotype derived from cell-cell adhesion controlled by a well-characterized pathway (*FLO* gene family members, and their transcriptional regulators). This pathway is activated in response to environmental cues including cell density, carbon and/or nitrogen sources, pH, temperature, oxygen, agitation, ethanol concentration, and the presence of cations (reviewed by [5,6]). Flocculation is undesirable during fed-batch bioethanol production because it impairs the centrifugation step required for cell recycling, and it also reduces the cell-substrate contact surface, thereby slowing fermentation kinetics and reducing yield [1,7].

Most strains used in sugarcane bioethanol production, including PE-2, are non-flocculant in pure culture. However, industrial-scale fermentations employing these strains occasionally exhibit flocculation-like features that cause significant productivity losses. In those cases, the flocculation-like phenotype is typically due to co-aggregation between yeast and bacterial contaminant cells [7,8], rather than conventional genetically determined yeast self flocculation. *Lactobacillus* species are the main bacterial contaminants found in sugarcane bioethanol production due to their ability to tolerate ethanol stress [8 - 11% (v/v)] and the anti-bacterial acid wash administered to the yeast cells prior to pitching each new batch (pH 2.0 – 3.0) [9]. *L. fermentum*, *L. vini* and *L. plantarum*, have been reported to be the main agents responsible for the co-aggregation of yeast cells [10,11]. The mannose-specific adhesin (Msa) found in *L. plantarum* and *L. fermentum* has been implicated in cell-cell interactions [12-14]. Hirayama *et al.* [15] examined co-aggregation in a panel of *S. cerevisiae* mutants with gene deletions of twelve mannan cell wall constituents. Among them, the *mnn2Δ* mutant strain lost the capacity to co-aggregate with *L. plantarum* cells. Mnn2p is a mannosyltransferase that transfers the first α-1,2-linked mannose to the mannan core structure to form a side chain that is subsequently extended by Mnn5p [16]. In the absence of Mnn2p, the mutant identified by Hirayama *et al.* [15] has an unbranched mannan chain that is incapable of linkage to the bacterial adhesin Msa.

Although *S. cerevisiae* is an acid-tolerant organism [17], exposure to high concentrations of organic acids produced by bacterial contaminants slows down the yeast metabolism and reduces fermentative fitness [18-20]. Narendranath *et al.* [21] reported that the synergism between lactic and acetic acids reduced the rates of yeast growth, glucose consumption, and ethanol production.

Although recent advances have been made in the characterization of the cellular pathways that contribute to the success of PE-2 as a bioethanol producer, such laboratory-based studies cannot accurately replicate the biotic and abiotic stresses encountered by this strain during industrial-scale fermentations [7,22]. To gain a better understanding of the molecular physiology of PE-2 under actual production conditions, we determined the gene expression profiles from cells collected directly from distilleries, and contrasted them to the transcriptional responses triggered by co-aggregation with bacterial contaminants. This dataset provides valuable information to support the genetic improvement of PE-2 and other bioethanol-producing strains, specifically, in the development of strategies to reduce or avoid co-aggregation in the presence of bacterial contaminants.

## Results and discussion

### Experimental dataset

Brazilian sugarcane bioethanol fermentation is characterized by a dynamic competition between high productivity industrial yeast strains and wild yeast and bacteria that contaminate the production process [1-3]. Early in the 2009 sugarcane harvest season (April), a pronounced flocculation-like phenotype was observed in the yeast population at a distillery in São Paulo state. Since the PE-2 yeast strain used as the initial inoculum is non-flocculant, the cell-cell adhesion observed was likely caused by co-aggregation between yeast cells and bacterial contaminants [7]. On that occasion, we collected samples directly from the fermentation tanks at seven time points of a single fed-batch cycle from this flocculated (FL) condition. The distillery’s operator initiated a combination of antibiotic and acid treatments to control the bacterial contamination and cellular co-aggregation. This treatment lasted for several weeks and was effective. By July, the culture had fully reverted to its original non-flocculated state (typical fermentation; TF), despite present similar number of bacteria (1 × 10^6^ CFU/mL). We then returned to the distillery and collected samples at six time points from the beginning to the end of a single disaggregated fed-batch cycle.

We isolated random yeast colonies from the FL and TF samples and genotyped them using PE-2 specific PCR markers recently developed by our group [2]. Despite the three-month interval between the collections, 95% of the yeast colony isolates from either flocculation condition matched the unique banding profile of the original PE-2 inoculum (data not shown). This result was consistent with the high degree of adaptation and persistence in long-term fed-batch sugarcane fermentation with cell recycling that is the hallmark feature of the PE-2 strain. It also reassured us that PE-2 was indeed the major yeast strain present in the microbial population, therefore allowing us to interpret the results of the transcriptomics studies presented below as a reflection of the molecular physiology of this particular strain.

### Alignment of RNA-seq reads and microbial identification

The RNA-seq libraries from the six TF and seven FL samples were sequenced using Illumina technology (see Methods). Combined, they totaled approximately 330 million 36-bp single-end reads and approximately 11.9 Gb of sequence information. On average, approximately 76% of the reads from each sample aligned to reference *S. cerevisiae* genes and were interpreted as being derived from PE-2 transcripts (Methods and Additional file 1).

**Figure 1:**
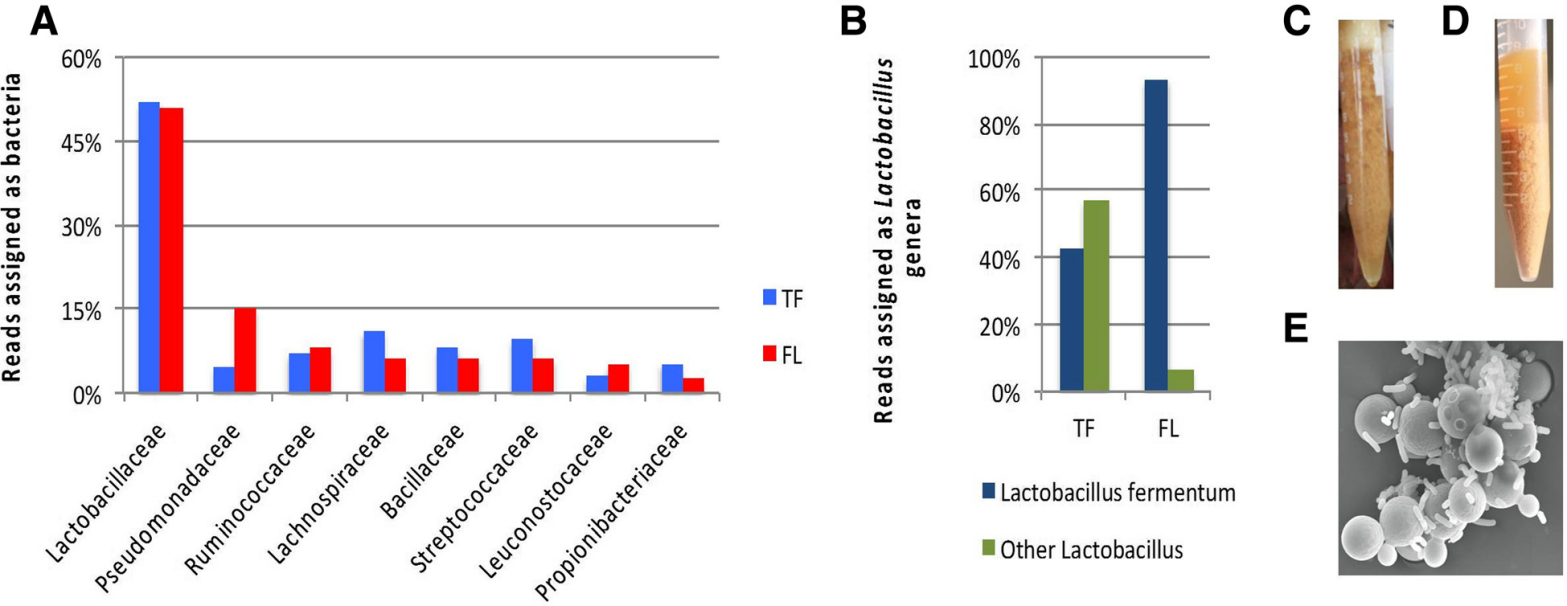
**Bacterial diversity during industrial fermentation. A**- Bacterial community represented by the family taxon level. The number of individuals from each family was obtained from the average number of reads identified for the time points of both conditions examined (TF – typical fermentation; FL – flocculated fermentation). **B**- The percentage of *L. fermentum* among the total *Lactobacillus* that were identified in the microbial community. The *Lactobacillaceae* family reads were subtracted from reads previously classified as bacteria in A. **C**- Picture taken at the time of sample collection in the plant. Flocs are under suspension due to high level of CO_2_ formed during fermentation. **D**- Illustration of flocculation assay at laboratory scale. **E**- Scanning electron micrograph showing co-aggregation between PE-2 yeast cells and *L. fermentum* at 5,000 times magnification. The image was captured after 30 hours of yeast and bacterium co-culture under laboratory conditions in **D**. Both microorganisms were isolated from the FL biological samples.

The material used to prepare the sequencing libraries also included some non-mRNA molecules, which were also sequenced and generated reads. We took advantage of this feature of the data and mined it for sequences derived from the bacterial cells present in the fermentations. We performed rRNA identification through alignment of the RNA-Seq reads to the SILVA rRNA database [23]. An average of approximately 5% of the total reads were classified as ribosomal sequences, with 0.26% being assigned to a bacterial origin (Additional file 1). The bacterial read counts per taxon were calculated for the different taxonomic levels using the SILVA rRNA database. The family level distribution of the bacterial sequences detected in the two fermentation conditions sampled are shown in Figure 1A. Interestingly, TF and FL had a similar overall distribution of bacterial families. However, within the *Lactobacillaceae* family, most of the reads derived from the flocculated condition were assigned to a single species, *Lactobacillus fermentum* (~93%) (Figure 1B). In contrast, only 41% of the *Lactobacillaceae* reads belonged to this species in the typical fermentations. This observation was significant since *L. fermentum* has been reported to induce sedimentation in *S. cerevisiae* [9,10]. To evaluate in principle the ability of *L. fermentum* to induce co-agregation with PE-2, we isolated bacterial colonies from this species from our FL samples and confirmed their identity by 16S rDNA PCR and Sanger sequencing. These isolates were co-cultured with PE-2 under laboratory conditions and a comparable behavior to that observed at the distillery was observed (Figure 1C and D). The PE-2 yeast cells became co-aggregated and sedimented when co-cultured with greater than 1 × 10^5^ *L. fermentum* cells/mL. A representative scanning electron micrograph of PE-2 yeast and *L. fermentum* bacterial cells from these co-cultures under laboratory conditions is shown in Figure 1E.

### Metabolite analysis

**Figure 2:**
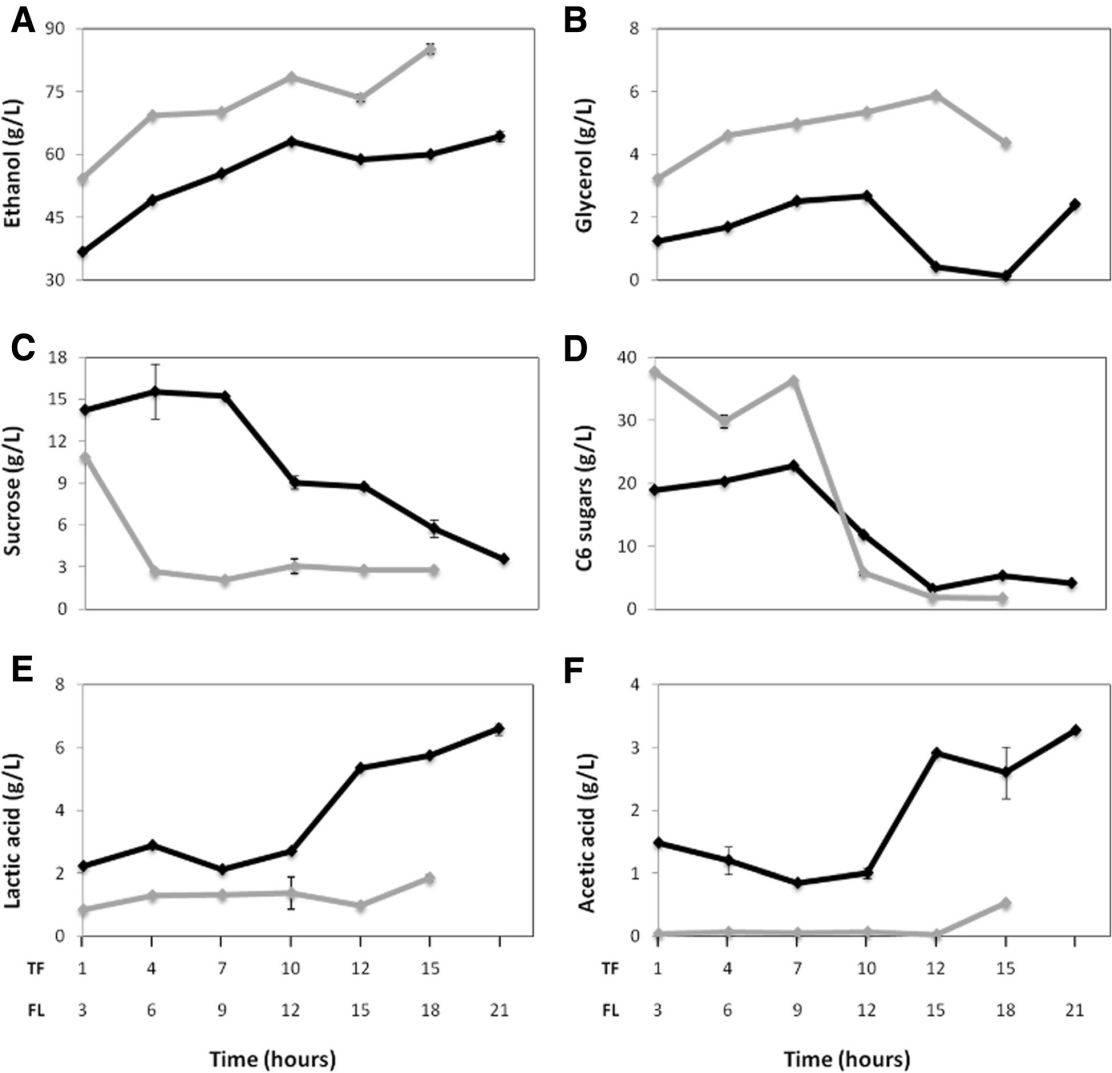
**Kinetics of production and consumption of major compounds examined during the fermentations.** Content in g/L of: **A**- ethanol; **B**- glycerol; **C**- sucrose; **D**- C6 sugars (glucose and fructose); **E**- lactic acid; and **F**- acetic acid. The compounds were measured with HPLC equipment using samples collected during the fermentations. FL - flocculated fermentation: black lines; TF - typical fermentation: gray lines. Standard deviation bars were obtained using three technical replicates for each time point. Please, note that the same time points between the two fermentation conditions (eg. FL3 vs. TF3) are not directly comparable in isolation, as they do not necessarily correspond to the same stage along each fermentation.

Analysis of the chemical composition of the collected samples revealed four significant differences in the kinetics of flocculated versus typical fermentation (Figure 2). Compared to the TF samples, the FL samples had (i) lower final ethanol titer, (ii) lower glycerol production, (iii) higher lactic and acetic acid concentrations, and (iv) slower rate of sucrose hydrolysis.

The fermentation batches took longer to complete in FL (21 hours) than in TF (15 hours), and the final ethanol content of the FL samples (64.4 g/L) was ~25% lower than that of the TF samples, although both had somewhat similar residual sugar levels (FL: 7.6 g/L; TF: 4.5 g/L).

Glycerol production by yeast cells is inversely associated with ethanol titer [1]; however, the production of a limited amount of glycerol is important to help maintain an optimal redox equilibrium and proper cellular osmoregulation [24,25]. One of the many desirable properties of PE-2 is its well-balanced production of glycerol, which is low enough to have high ethanol yield, but high enough to tolerate stress. Interestingly, TF samples had almost three times higher glycerol concentration (4.74 g/L) than FL, suggesting that the FL cells were metabolically imbalanced and thus were likely less tolerant to the stressful environment of industrial fermentation.

*Saccharomyces cerevisiae* strains do not produce high amounts of organic acids [17,26], therefore most organic acids detected during bioethanol production have been attributed to bacterial contaminants, primarily *Lactobacillus* [9,27]. Under laboratory conditions, PE-2 produces only 1.5 and 2.4 mg/L of lactic and acetic acids, respectively [18]. In our study, the final content of acetic and lactic acids was 6 and 3.5 fold higher, respectively, in FL relative to TF (Figure 2). Since the overall residual sugar contents were similar at the end of both fermentation conditions, the flow of the sugar feedstock must have been significantly altered in the FL fermentation condition, being diverted from the intended ethanol production by yeast to instead being misused by bacterial contaminants to produce organic acids.

Due to its prolonged duration and the low ethanol titer, we estimated that co-aggregated fermentation resulted in the loss of approximately 12 million liters of bioethanol during the three months that the distillery operated under this condition, underscoring the critical importance of this problem to the bioethanol industry.

### Differential gene expression analysis

The number of reads from the respective RNA-seq libraries that aligned to reference genes was used in the identification of differentially expressed (DE) genes between the time courses of the two fermentation conditions, as well as within each of the two conditions (Table 1). Time points TF1 and FL1 were used as references. Gene expression comparisons between fermentations (Table 1, C- Comparative) were performed by comparing time points from early phases of fermentation (*e.g.* TF1 vs. FL1) and late stages (*e.g.* TF6 vs. FL7). Moreover, we performed a global analysis using all TF libraries versus all FL libraries (TFs vs. FLs) to examine the cumulative effect of all time points for each fermentation condition. DE genes were obtained from the global analysis using gene expression averages from the six time-points of TF compared with the seven time-points of FL, with a p-value cutoff of 0.01 (Table 1). The complete RNA-seq data are available at the Gene Expression Omnibus (http://www.ncbi.nlm.nih.gov/geo) under accession number [GSE41834] (Additional file 1), and the lists of DE genes are shown in Additional files 2, 3 and 4.

**Table 1:** **Differentially expressed genes during industrial bioethanol fermentation under two distinct conditions**

**A- Typical fermentation**	**DE genes**	**Down-regulated genes**	**Up-regulated genes**
TF1 vs. TF2	305	165	140
TF1 vs. TF3	989	577	412
TF1 vs. TF4	1506	554	952
TF1 vs. TF5	1609	581	1028
TF1 vs. TF6	2396	1588	808
**B- Flocculated fermentation**			
FL1 vs. FL2	353	261	92
FL1 vs. FL3	679	518	161
FL1 vs. FL4	1847	1278	569
FL1 vs. FL5	3412	2882	530
FL1 vs. FL6	3518	3034	484
FL1 vs. FL7	3735	3263	472
**C- Comparative**			
TF1 vs. FL1	603	95	508
TF6 vs. FL7	1473	1255	218
TFs vs. FLs	390	274	116

Genes were considered differentially expressed (DE) if they had an expression ratio ≥2 or ≤ -2 and a p < 0.01. Down-regulated genes included TF1 (A), FL1 (B) and TF1, TF6 and TFs (C). Up-regulated genes included TF2-TF6 (A), FL2-FL7 (B) and FL1, FL7 and FLs (C).

**Figure 3:**
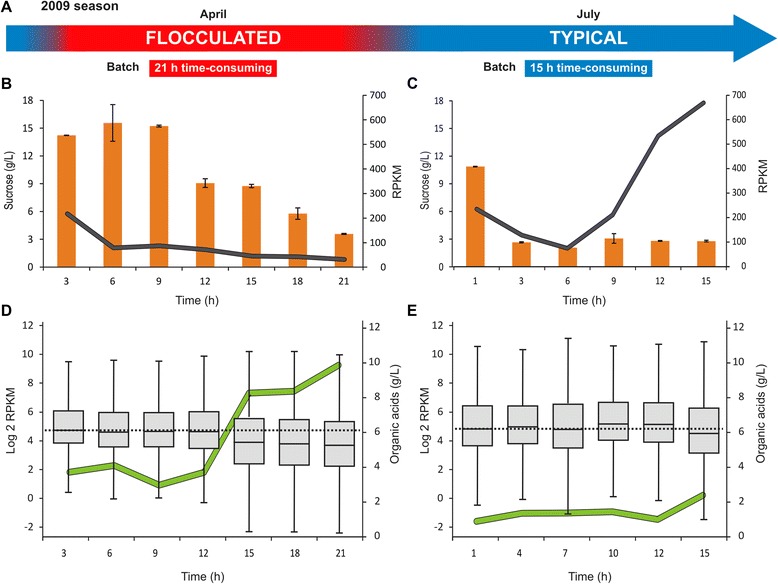
**Global gene expression distribution and its correlation with the content of organic acids. A**- Schematic representation of the fermentation conditions found at the Nova América distillery during the 2009 season. **B** and **C**- Transcriptional profile of *SUC2* gene described by RPKM metric (black lines) and sucrose concentration (orange bars) in function of fermentation time for FL **(B)** and TF **(C)** conditions. **D** and **E**- Boxplot of the log2 RPKM for the flocculated **(D)** and typical **(E)** fermentations. Dotted lines represent the median gene expression value of the samples. The concentration of organic acids (green line) was obtained by the sum of the lactic and acetic acid contents identified for each time-point.

Despite the lower content of ethanol obtained in the two conditions, glycolysis-related genes were not differentially expressed between them. Curiously, the sucrose-hydrolyzing gene, *SUC2*, was up-regulated by a factor of 4 in the TF condition. Furthermore, the expression of *SUC2* in the TF condition increased three-fold when the addition of sugarcane extract stopped (TF3) and the level of C6 sugars (glucose and fructose) was reduced from 36 g/L to 6 g/L. Figure 3 shows the transcriptional profile of *SUC2*, described by the RPKM metric and sucrose concentration, as a function of fermentation time for the FL (3B) and TF (3C) conditions. This rapid activation of *SUC2* expression appears to be important for the prompt stress response to nutrient limitation (*i.e.* C6 sugars) during fermentation [28]. However, *SUC2* expression in the FL samples decreased seven-fold during the process. This pattern could be partially explained by the presence of sucrose in the FL samples during the entire fermentation process (Figure 2C), which would have provided a continuous supply of C6 sugars to the cells and might have thus caused *SUC2* repression [29]. The reduced surface contact between yeast cells and the medium due to cell-cell adhesion in FL could be associated with this distinct pattern of sucrose consumption. Therefore, we can reason that one of the causes of the longer time required for the FL fermentation could be the down-regulation of *SUC2*, leading to a low availability of fermentable sugars.

Although *S. cerevisiae* is a vigorous and acid-tolerant fermentative organism [17], high concentrations of organic acids with a low pH and high concentration of ethanol reduce its metabolic rate [18-20]. We determined the pH for the FL and TF samples and obtained comparable measurements ranging from pH 3.8 to pH 4.3. As shown in Figure 3D, the overall levels of gene expression decreased when the organic acid content reached values greater than 4 g/L in the FL samples, suggesting that organic acids produce strong gene repression in the yeast cells. In this case, fewer genes were expressed and at lower levels compared with the previous time-points. At the low pH of fermentative conditions, the organic acids produced by contaminating bacteria exist primarily in their undissociated state [30]. Such undissociated organic acids present in the substrate diffuse across the cell membrane and dissociate in the cytoplasm, generating protons that lower the intracellular pH and inhibit many metabolic functions [31]. Moreover, this dissociation produces charged anions that can in turn produce free radicals, leading to severe oxidative stress [31]. Narendranath *et al.* [21] reported a negative synergist effect between lactic and acetic acids when concentrations of organic acids are present in the medium at 0.5% (w/v) and 0.04% (w/v), respectively. This combination inhibited the cellular growth rate and decreased the rates of glucose consumption and ethanol production.

**Figure 4:**
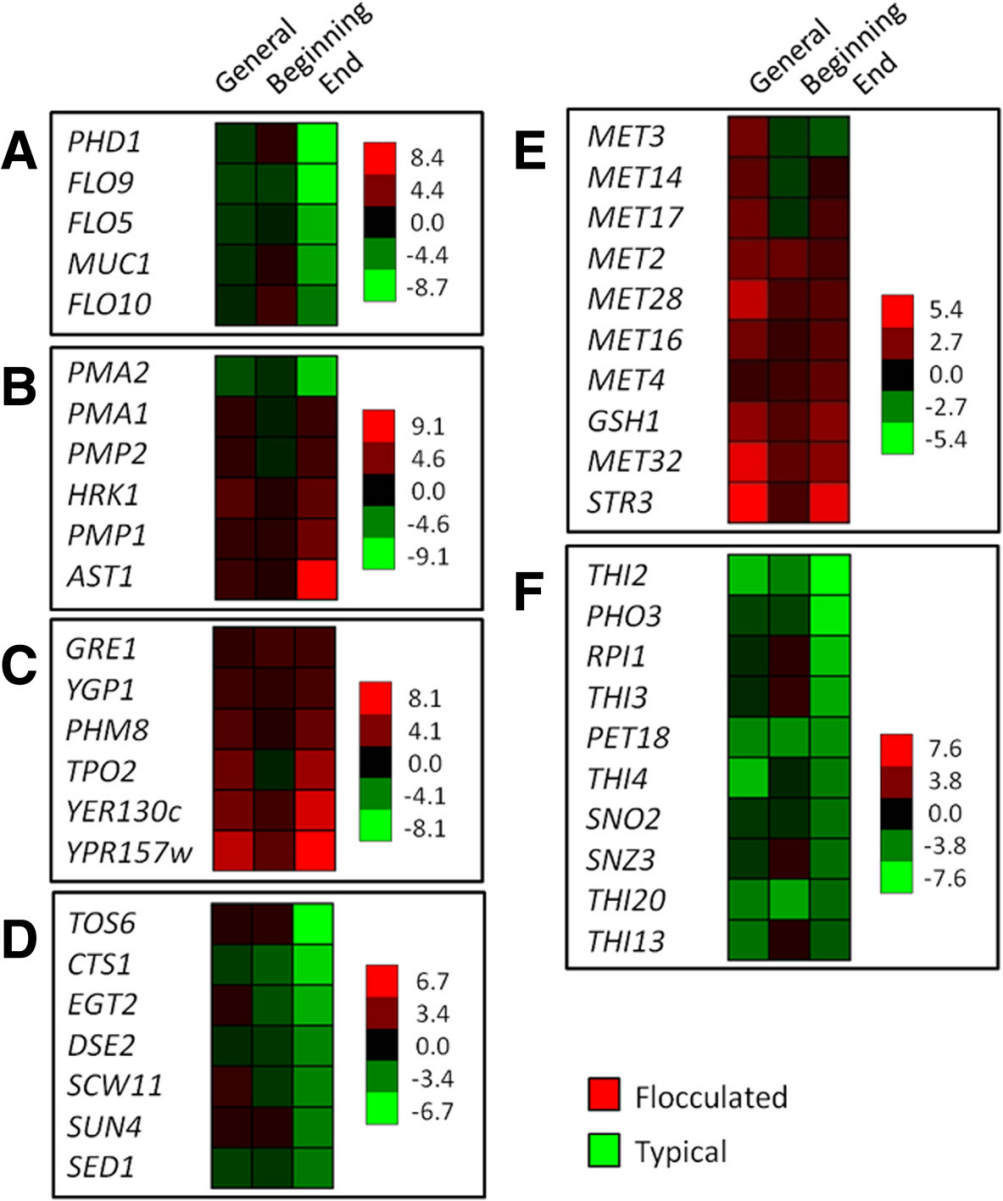
**Gene expression comparisons between typical and flocculated fermentations. A**- FLO genes and flocculation activators; **B**- Plasma membrane H^+^-ATPase (*PMA1*) and related genes; **C**- Haa1p target genes; **D**- Cell wall components; **E**- Methionine- and glutathione-related genes; **F**- Thiamine metabolic process genes. Differentially expressed (DE) genes were defined as those with a fold change ≥2 or ≥ -2 and a p-value <0.01. Negative values were obtained for the TF samples, and positive values were obtained for the FL samples. General analysis (TFs vs. FLs) was performed using six time-points for the TF samples and seven time-points for the FL samples. The beginning of fermentation is denoted as TF1 and FL1, and the end of fermentation is denoted as TF6 and FL7. The software Expander6 was used for the gene clustering image drawn using the end of fermentation as a reference.

Genes related to flocculation (*MUC1, FLO5, FLO8, FLO9, FLO10* and *PHD1*) were not found to be up-regulated in the FL samples (Figure 4A). This result confirmed that the observed cellular co-aggregation was not due to yeast genetic control. We observed that the main transcriptional differences between the FL and TF conditions were related to content variations in the concentrations of organic acids present in the medium. The major plasma membrane H^+^-ATPase, encoded by *PMA1* [32], was not differentially expressed between samples at the beginning of fermentation (TF1 vs. FL1). However, we verified a two-fold *PMA1* induction in flocculated fermentations at the last time point. Pma1p-related genes, *AST1* (targeting factor to plasma membrane), *PMP1, PMP2* and *HRK1* (regulatory elements), had similar expression patterns (Figure 4B). These data show that the mechanism used to pump out protons to regulate cytoplasmic pH is up-regulated in the FL cells. This stress response, however, consumes excessive ATP and may cause an inhibitory action by energy depletion [31].

Previous studies have shown that the main transcriptional responses of *S. cerevisiae* in the presence of weak acids (lactic and acetic) are related to cell wall components, membrane-associated transport processes and iron homeostasis [19,33,34]. The *HAA1* transcription factor and Haa1p-regulated genes have been reported to be up-regulated in response to lactic and acetic acids [34-37]. Among the Haa1p target genes, we observed the up-regulation of *TPO2*, *YGP1*, *PHM8*, *GRE1*, *YPR157w*, *YER130c* and *HRK1* in the FL7 sample compared with TF6 (Figure 4C). However, we did not observe differences in the expression of *HAA1* itself between fermentations, suggesting a co-regulation of those seven genes by distinct transcription factors [36].

During FL, cell wall-related genes changed their expression dramatically compared with TF. Kawahata *et al.* [19] reported that the depleted expression of the cell wall components *SED1*, *DSE2*, *CTS1*, *EGT2*, *SCW11*, *SUN4* and *TOS6* increased the resistance of *S. cerevisiae* to lactic acid. Here, the PE-2 strain used the same mechanism for FL, down-regulating these seven genes by a factor of 3- to 6.8-fold in response to the organic acid concentrations at the end of the FL time course (Figure 4D).

To validate the RNA-seq data, 15 genes were assessed by RT-qPCR, for a total of 60 pairwise comparisons. The total expression trends of the time-points analyzed were 87% similar between the different techniques, with correlation values of R^2^ = 0.7604 and R^2^ = 0.7951 for the FL and TF samples, respectively (Additional file 5).

### Gene ontology of DE genes

Gene ontology (GO) analyses were performed to identify functional signatures in gene expression using the DE genes between fermentation conditions (TF1 vs. FL1; TF6 vs. FL7; TFs vs. FLs). Two enriched GO terms were particularly meaningful in the context of industrial fermentations: cellular amino acid and vitamin metabolic processes (Additional file 6).

For the FL samples, several genes assigned as “cellular amino acid metabolic process” (*MET2*, *MET3*, *MET4*, *MET14*, *MET16*, *MET17*, *MET28*, *MET32*, *STR3* and *GSH1*) are involved mainly in the methionine (MET) and glutathione (GSH) biosynthesis pathways (Figure 4E). GSH has an important role in the protection of *S. cerevisiae* against oxidative stress [38,39]. The first, and rate-limiting, step in the GSH biosynthetic pathway occurs when *GSH1* catalyzes the conjugation of glutamate and cysteine (reviewed in [40]). Because methionine is involved in cysteine biosynthesis, the expression profile of the *MET* genes has a direct effect on GSH biosynthesis by supplying cysteine to the pathway [41]. Moreover, the transcription factors for Met4p and Met32p, which are required expression of *MET* biosynthetic genes, are also essential for *GSH1* expression by cadmium-mediated regulation [42]. The MET- and GSH-related gene expression profiles (up-regulated at FL) suggest that the yeast cells in the FL samples were under oxidative stress, most likely due to the formation of intracellular reactive oxygen species triggered by lactic [43] and acetic [44] acids.

Genes involved in the vitamin-related metabolic process were identified prominently up-regulated in the TF samples. Interestingly, most of the genes identified (*e.g.*, *PET18*, *PHO3*, *RPI1*, *THI2*, *THI3*, *THI4*, *THI13*, *THI20*, *SNO2* and *SNZ3*) participate in thiamine (vitamin B1) metabolic processes (Figure 4F). The *SNO/SNZ* genes are required for vitamin B1 and B6 biosynthesis and also have a role in oxidative stress tolerance [45-47]. Moreover, under laboratory conditions, bioethanol strains carrying amplifications of these genes have been shown to be less sensitive to fluctuation in the vitamin B levels when cultured in a medium with a high sugar concentration [48], and these genes have been suggested to be important for adaptive growth in an industrial process [4,48]. Our transcriptional data is consistent with those reports and underscores the importance of thiamine genes for the adaptation of the PE-2 strain to sugarcane bioethanol production.

### Differential allelic expression

Sequencing analysis of the PE-2 genome revealed that this strain is highly heterozygous [4]. We took advantage of the high number of PE-2 sequences generated by the RNA-seq reads (~9 Gb) to identify differences in allelic expression during the different fermentations. Differential allelic expression (DAE) at a threshold of 2-fold between alleles was used for a case of DAE to be called (*i.e.*, more than 66% of the reads aligned to a specific gene came from a single allele). When the coding region had more than one heterozygous SNP, DAE was determined accounting for the cumulative imbalance for all the phased SNPs across the entire gene.

**Figure 5:**
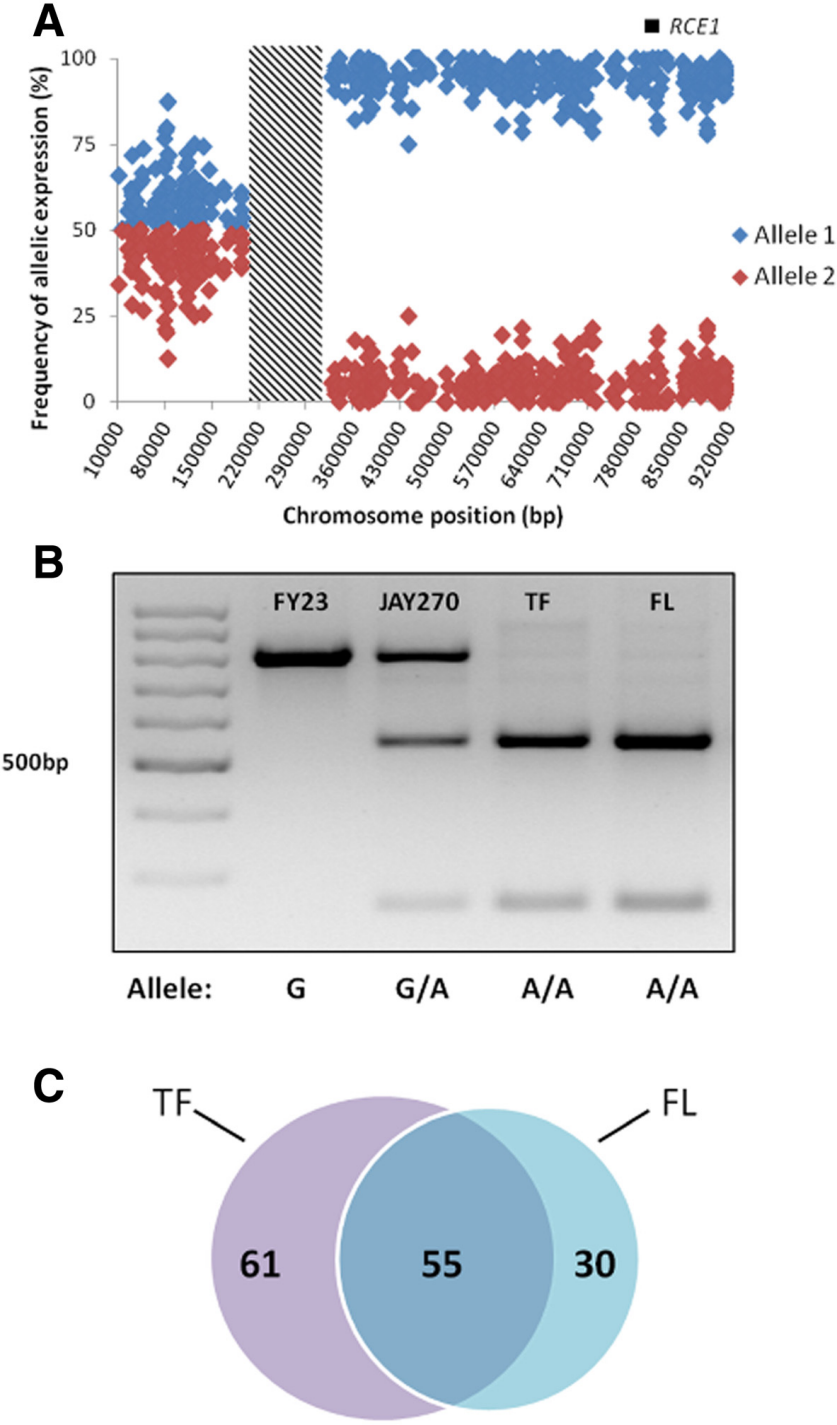
**Analysis of differential allelic expression (DAE). A**- Allelic expression frequency of the genes located on chromosome 13 (Chr13). The allele with higher expression was arbitrarily designated as allele 1 (blue) and the lower expression allele was designated as allele 2 (red). The hatched box represents a 100-kb homozygous region (including the centromere) in the JAY270/PE2 diploid where no DAE information could be assessed. The DAE plot suggests that the breakpoint of the Chr13 LOH event occurred within the homozygous region. The position of the *RCE1* locus on the right arm is shown. **B**- The genotypes at the *RCE1* locus were determined by PCR followed by restriction analysis using *Mbo*I. The predicted banding patterns for the alleles were: Homozygous for allele 1 (cut) 543 bp and 256 bp; Homozygous for allele 2 (uncut) 799 bp; Heterozygous pattern: 799 bp, 543 bp and 256 bp. A molecular weight marker ladder of 100 bp incremental size fragments was used in line 1. The 500 bp marker band is indicated. **C**- Venn diagram showing the number of DAE genes identified exclusively in the TF and FL, and the DAE genes identified in simultaneously in both TF and FL.

Our initial analysis identified 195 candidate DAE genes that were found in both TF and FL conditions (Additional file 7; TF and FL). Interestingly, 140 of those genes were located at consecutive positions on the right arm of Chr13, between *FAR8* (*YMR029c*) and the right telomere (*TEL13R*), and all of them had reads that were essentially derived from only one allele (Figure 5A). This striking pattern suggested that a Chr13 region of approximately 600 kb was homozygous in the PE-2 strain present at the time in the distillery, likely due to a mitotic recombination event leading to loss of heterozygosity (LOH). To confirm this homozygous pattern in the industrial isolates, we designed primers to partially amplify the *RCE1* (YMR274c) locus, which contains a heterozygous SNP (A/G) in the JAY270/PE-2 strain at position 874 within a recognition site for the *Mbo*I restriction endonuclease. *RCE1* sequences were amplified from PE-2 cells isolated from the industrial fermentations as well as from the JAY270/PE-2 heterozygous diploid and from the S288c isogenic strain FY23 (uncut allele). The *Mbo*I digestions of the PCR products confirmed that the PE-2 cells isolated from industrial tanks were indeed homozygous for *RCE1*, while a heterozygous pattern was observed for JAY270 (Figure 5B).

Exclusion of the Chr13 right arm genes resulted in 55 genes with DAE identified in both fermentations. In addition, we also found 61 DAE genes exclusively in the TF samples, and 33 exclusively in the FL samples (Figure 5C and Additional file 7), suggesting a fermentation condition-dependent expression pattern regulated by specific transcriptional responses. We hypothesize that the observed DAE patterns may be due to the differential effect on allele expression of heterozygous SNPs at *cis*-elements at the regulatory regions of the DAE genes. Gene ontology analysis of the DAE genes did not reveal a functional enrichment in this relatively limited gene set (the only statistically enriched GO term detected was “unknown function”). Although individual cases of DAE may play an important role in dictating the fermentation performance of PE-2 under typical and/or co-aggregated conditions, it is unclear at this point which are those genes and what is their specific function.

## Conclusion

The gap in the basic biological knowledge about PE-2 and its related strains represents a significant barrier to genetically improving these strains and fully exploiting their biotechnological potential [8]. The genetic engineering of bioethanol strains should be preceded by genomic and transcriptomic studies to identify the genetic characteristics that are associated with yeast fermentative fitness [7,22]. The results presented here provide new insights into the biology of the PE-2 strain and allowed us to identify stress response mechanisms during bioethanol production. Information derived directly from industrial scale fermentations can be used to support studies aimed at developing superior fermentative fitness in the PE-2 strain. The data described here represent an important step to reach those goals.

## Methods

### Fermentation samples collection

Biological samples from two different industrial fermentation conditions were collected directly from bioethanol fermentation tanks at the Nova América distillery (Maracaí-SP, Brazil). At the beginning of the 2009 season, the PE-2 culture used as the starting inoculum at this distillery became flocculent due to bacterial co-aggregation. On that occasion, we collected samples at seven different fermentation time points (FL samples) during one fed-batch cycle. Over the following three months, the yeast cells were treated with antibiotics and sulfuric acid before re-pitching the next batch. The yeast community eventually reverted to its typical phenotype (disaggregated), at which time samples were collected at six intervals during a typical fed-batch fermentation cycle (TF samples). Three biological replicates were collected for each of the thirteen sampled time-points. After collection, the samples were immediately transferred to a container with dry ice for the posterior chemical and transcriptomic analysis. Aliquots of each sample were also maintained on ice to preserve the viability of cells.

### Yeast genotyping

Unfrozen aliquots from each condition (FL and TF) were diluted and plated in YPD solid medium (yeast extract 10 g/L [w/v], peptone 20 g/L [w/v], glucose 20 g/L [w/v] and agar 20 g/L [w/v]). Colonies were isolated and DNA extraction was performed following to a phenol-chloroform protocol [49]. Twenty yeast colonies derived from each condition were analyzed using PE-2 specific PCR markers as described by Carvalho-Netto *et al*. [2].

### Metabolite analysis

Aliquots of the biological replicates were centrifuged, and the supernatants were diluted in water (1:3), filtered in Millipore 0.22-μm filters, and analyzed by High Performance Liquid Chromatography (Alliance 2795, Waters, Milford, MA, USA) using a refractive index detector (HPLC-RI) and an Aminex HP-87H column (Bio-Rad Laboratories, Hercules, CA, USA). The HPLC readings for sucrose, glucose, fructose, ethanol, glycerol, acetic acid and lactic acid in the samples were fit to respective standard curves to determine their concentrations.

### RNA isolation and RNA-seq library preparation

The total RNA of the samples was extracted using a phenol and chloroform protocol [50]. Illumina RNA-seq libraries were prepared following the manufacturer’s recommendations. Briefly, mRNA was isolated from 1 μg of the total RNA using oligo(dt) magnetic beads, and then fragmented in the presence of divalent zinc ions. The fragmented RNA was then used for first and second strand cDNA synthesis. Double-stranded cDNA was end-repaired and 3’ adenylated for the ligation of sequencing adapters. After adapter ligation, fragments of approximately 250 bp were isolated by gel electrophoresis and PCR amplified. The libraries were validated on an Experion DNA chip (Bio-Rad, Hercules, CA, USA) and quantified using a Qubit fluorometer (Invitrogen, Carlsbad, CA, USA). Each library was sequenced in one lane of an Illumina Genome Analyzer II× (GAII×) sequencer, resulting in 20-30 million 36-bp single-end reads.

### Gene expression analysis and functional annotations

The complete dataset of RNA-seq reads has been deposited in SRA under accession number [SRA057038]. For each RNA-seq library, reads were aligned to a custom reference gene database constituted by *S. cerevisiae* S288c genes (www.yeastgenome.org) and 20 JAY291-specific genes [4] (Additional file 1). The alignment was performed using SOAPaligner version 2.20 [51], allowing up to two base mismatches and discarding repeat reads. A Perl script was then created to calculate the number of reads aligned per gene for each RNA-seq library.

The output file was analyzed using the DEGseq package [52] for the identification of differentially expressed (DE) genes. Pairwise comparisons within a fermentation condition (typical and flocculated, individually) and between fermentation conditions (typical versus flocculated) are shown in Table 1. For the comparative analysis (TFs vs. FLs), the DEGseq was configured to use the time points within fermentation (TF1-TF6 and FL1-FL7) as experimental replicates. A p-value cutoff of 0.01, with a fold change > 2 (up-regulated) or < -2 (down-regulated), were used to determine the DE genes in these comparisons. Gene expression levels were defined using the RPKM formula [53]. Clustering and visualization of the DE genes were obtained using EXPANDER [54].

We also quantified the genomic background transcription (RPKM threshold) using 1787 intergenic regions larger than 500 bp. The RPKM threshold was estimated through the alignment of reads to intergenic regions using SOAPaligner [51], allowing up to two base mismatches and discarding all repeat reads. The distribution of the RPKM values from the genes and intergenic regions for each RNA-seq library was used to estimate the RPKM threshold by visual inspection. The gene expression levels with RPKM values below the RPKM threshold were not considered to be expressed genes, and these genes were discarded from the differential expression analysis when the expression levels were below the RPKM threshold in the respective libraries.

Gene ontology (GO) terms of the DE genes were obtained from SGD (http://www.yeastgenome.org/cgi-bin/GO/goSlimMapper.pl) using the Yeast GO-Slim Process parameters and a cutoff p-value <0.01. Functional GO enrichment terms were obtained using DE genes between fermentations (TF1 vs. FL1; TF6 vs. FL7; TFs vs. FLs).

### RNA-seq validation by Real Time qPCR (RT-qPCR)

To confirm the RNA-seq results, 15 genes were analyzed by RT-qPCR in four pairwise comparisons (TF1 vs. TF4, TF1 vs. TF6, FL1 vs. FL4 and FL1 vs. FL7), for a total of 60 pairwise comparisons. A list of the genes and primers used is presented in Additional file 8. Aliquots of the samples used to construct the RNA-seq libraries were used in transcriptase reverse reactions to synthesize cDNA using the *SuperScript Direct cDNA Labeling System* (Invitrogen, Carlsbad, CA, USA) according to the procedures described by the manufacturer. The RT-qPCR mix consisted of 8 μL of SYBR Green Supermix (Bio-Rad Laboratories), 1 μL of each primer (0.5 μM final concentration), 5 μL of water and 1 μL of cDNA. The reaction program consisted of one hold at 95°C for 5 min, followed by 40 cycles of 15 s at 95°C and 75 s at 60°C. Fragment amplification and detection of *SYBR Green* (Applied Biosystems, CA, USA) were performed with the *Step One Plus* thermalcycler (Applied Biosystems). The relative expression ratio was calculated using the 2^-ΔΔCT^ method [55] using primers with amplification efficiencies between 90–100% (−3.6 ≥ slope ≥ −3.3). The *ACT1* and *YNL134c* genes were selected as endogenous genes to normalize the expression values for the TF and FL samples, respectively, as both genes showed little variation in expression among the different RNA-seq libraries.

### Bacterial identification

Although conventional RNA-seq libraries are enriched for mRNA through the use of oligo(dt) magnetic beads, a small proportion of sequences corresponding to other RNA species is often detected [56], allowing the identification of the bacterial species present in the FL and TF samples. The RNA-seq reads were aligned into the SILVA rRNA database [23] using SOAPaligner, configured to allow two mismatches and discard any repeat reads. A custom Perl script was developed to parse the output file obtaining the read counts per taxon using different taxonomic levels. Bacterial families that accounted for less than 5% of the total reads from the TF or FL samples were not used in further analyses.

To identify the bacterial species associated with yeast co-aggregation, bacterial colonies were isolated in LB solid medium (tryptone 10 g/L [w/v], yeast extract 5 g/L [w/v], NaCl 10 g/L [w/v] and agar 20 g/L [w/v]) under anaerobic conditions. The bacterial DNA extraction protocol was adapted from Collart and Oliviero [50] using lysozyme (100 mg/mL) and proteinase K (10 mg/mL). PCR amplification of the 16S rDNA was performed in a final volume of 50 μL. The reaction mix consisted of 4 ng of DNA, 0.5 μM each of F27 (5′ AGA GTT TGA TCM TGG CTC AG 3′) and R1378 (5′ CGG TGT GTA CAA GGC CCG GGA ACG 3′) primers [57], 0.25 mM each dNTP, 3.5 mM MgCl_2_, 1X Colorless GoTaq Flexi Buffer (Promega, Madison, WI, USA) and 1.25 U of GoTaq Flexi DNA Polymerase (Promega). The amplification program consisted of one initial hold at 94°C for 3 min, followed by 40 cycles of 30 s at 94°C, 30 s at 55°C and 60 s at 72°C. A final 5-min extension was performed at 72°C. The PCR products were purified using the NucleoSpin Extract II purification kit (Macherey-Nagel, Düren, Germany), according to the manufacturer’s instructions. The PCR products (45 ng) were Sanger-sequenced using the Big Dye Terminator kit (Applied Biosystems, Foster City, CA, USA) on a 3500 Genetic Analyzer (Applied Biosystems). The reaction program consisted of one hold at 94°C for 2 min, followed by 35 cycles of 20 s at 94°C, 30 s at 55°C and 2 min at 60°C. Bacterial rDNA sequence similarity was obtained by BLASTn analysis using the GenBank non-redundant (NR) Database (http://blast.ncbi.nlm.nih.gov/).

### *RCE1* amplification and genotyping

For the loss of heterozygosity (LOH) analysis, a segment of the *RCE1* gene was PCR-amplified in a final volume reaction of 50 μL using 1 ng of yeast genomic DNA, 0.5 μM each of RCE1_F (5′ ACC TTA TAT TGT GGA CCC GTT 3′) and RCE1_R (5′ CTC GAT AGA ATT CCA TAA TAG 3′) primers, 0.25 mM each dNTP, 3.5 mM MgCl_2_, 1X Colorless GoTaq Flexi Buffer and 1.25 U of GoTaq Flexi DNA Polymerase (Promega, Madison, WI, USA). The amplification program consisted of one hold at 94°C for 2 min, followed by 35 cycles of 40 s at 94°C, 40 s at 56°C and 80 s at 72°C. A final 5-min extension was performed at 72°C. The PCR products were purified and digested using 10 U of *Mbo*I (New England Biolabs, Ipswich, MA, USA). The digested fragments were resolved in 2% (w/v) agarose gels and visualized by ethidium bromide staining.

## Additional files

Additional file 1: Table S1. Reads obtained by RNA-seq analysis during industrial bioethanol production. For each RNA-seq library, reads were aligned to a custom reference gene database constituted by *S. cerevisiae* S288c genes (www.yeastgenome.org) and 20 JAY291-specific genes. In order to assigned ribosomal sequences, reads were aligned into the SILVA rRNA database. Additional file 2: Table S2. Differentially expressed genes among the fermentations. Pairwise comparisons between fermentation conditions (typical versus flocculated) were performed using the time points within fermentation (TF1-TF6 and FL1-FL7) as experimental replicates. A p-value cutoff of 0.01, with a fold change > 2 (up-regulated) or < -2 (down-regulated), were used to determine the differentially expressed (DE) genes in this comparison. Gene expression levels were defined using the RPKM formula. Additional file 3: Table S3. Differentially expressed genes during flocculated fermentation. Pairwise comparisons within flocculated fermentation time points were performed using FL1 sample as reference. A p-value cutoff of 0.01, with a fold change > 2 (up-regulated) or < -2 (down-regulated), were used to determine the differentially expressed (DE) genes in these comparisons. Gene expression levels were defined using the RPKM formula. Additional file 4: Table S4. Differentially expressed genes during typical fermentation. Pairwise comparisons within typical fermentation time points were performed using TF1 sample as reference. A p-value cutoff of 0.01, with a fold change > 2 (up-regulated) or < -2 (down-regulated), were used to determine the differentially expressed (DE) genes in these comparisons. Gene expression levels were defined using the RPKM formula. Additional file 5: Figure S1. Correlation between RNA-seq and rt-qPCR data to fifteen selected genes. A- Flocculated fermentation (FL); B- Typical fermentation (TF). Samples TF1 and FL1 were used as references in order to obtain expression ratio among TF4 and TF6 and FL4 and FL7 samples, respectively. Expression values were obtained using three techniques replicates and are presented as fold change Log2. Additional file 6: Table S5. Enriched GO terms between fermentations. Gene ontology (GO) terms of the differentially expressed (DE) genes were obtained from SGD (http://www.yeastgenome.org/cgi-bin/GO/goSlimMapper.pl) using the Yeast GO-Slim Process parameters and a cutoff p-value <0.01. Functional GO enrichment terms were obtained using DE genes between fermentations (TF1 vs. FL1; TF6 vs. FL7; TFs vs. FLs). FL: Flocculated fermentation. TF: Typical fermentation. Additional file 7: Table S6. Genes with differential allelic expression. Differential allelic expression (DAE) was determined using a threshold of 2-fold between alleles, *i.e.*, more than 66% of the reads aligned to a specific gene came from a single allele. When the coding region had more than one heterozygous SNP, DAE was determined accounting for the cumulative imbalance for all the phased SNPs across the entire gene. DAE was identified in both fermentations (TF and FL), exclusively in TF and exclusively in FL. FL: Flocculated fermentation. TF: Typical fermentation. Additional file 8: Table S7. List of genes, PCR products, and primers used in rt-qPCR analysis.

## Abbreviations

Chr: Chromosome
DAE: Differential allelic expression
DE: Differential expression
GSH: Glutathione
Kb: Kilobase pairs
LOH: Loss of heterozygosity
MET: Methionine
RT-qPCR: Reverse transcriptase quantitative polymerase chain reaction
SNP: Single nucleotide polymorphism
