# Supplementary material for: Saccharomyces cerevisiae transcriptional reprograming due to bacterial contamination during industrial scale bioethanol production
Source: Microb Cell Fact. 2015 Jan 30;14:13. doi: 10.1186/s12934-015-0196-6 (PMC4318157; doi:10.1186/s12934-015-0196-6)
Supplement: Additional file 1: Table S1. — Reads obtained by RNA-seq analysis during industrial bioethanol production. For each RNA-seq library, reads were aligned to a custom reference gene database constituted by S. cerevisiae S288c genes (www.yeastgenome.org) and 20 JAY291-specific genes. In order to assigned ribosomal sequences, reads were aligned into the SILVA rRNA database. [file 12934_2015_196_MOESM1_ESM.docx]

**Additional file 1.**

| Time point (hours) | Total reads | Genome mapped reads (%) | Eukaryota ribosomal mapped reads (%) | Bacteria ribosomal mapped reads (%) |
| --- | --- | --- | --- | --- |
| TF1 (1) | 21,846,994 | 63.20 | 13.68 | 0.28 |
| TF2 (4) | 27,363,094 | 57.84 | 14.96 | 0.57 |
| TF3 (7) | 26,447,084 | 53.03 | 13.29 | 0.98 |
| TF4 (10) | 24,102,684 | 85.19 | 1.19 | 0.08 |
| TF5 (12) | 18,632,984 | 83.82 | 1.36 | 0.04 |
| TF6 (15) | 19,710,705 | 80.14 | 1.01 | 0.05 |
| FL1 (3) | 27,842,955 | 83.84 | 1.60 | 0.12 |
| FL2 (6) | 27,271,456 | 80.18 | 2.81 | 0.41 |
| FL3 (9) | 30,057,838 | 84.23 | 1.29 | 0.14 |
| FL4 (12) | 26,170,783 | 81.92 | 0.80 | 0.12 |
| FL5 (15) | 24,027,846 | 77.40 | 2.21 | 0.13 |
| FL6 (18) | 29,783,524 | 77.68 | 3.70 | 0.22 |
| FL7 (21) | 27,055,657 | 74.67 | 3.62 | 0.25 |
| Average | 25,408,738 | 75.63 | 4.73 | 0.26 |
| TF: typical fermentation; FL: flocculated fermentation. | | | | |
