# Supplementary figures and images for: Saccharomyces cerevisiae transcriptional reprograming due to bacterial contamination during industrial scale bioethanol production
Source: Microb Cell Fact. 2015 Jan 30;14:13. doi: 10.1186/s12934-015-0196-6 (PMC4318157; doi:10.1186/s12934-015-0196-6)

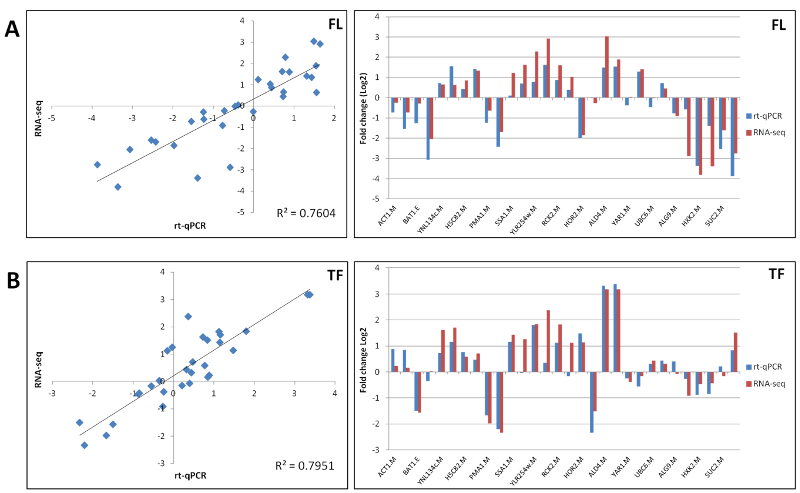

Supplement: Additional file 5: Figure S1. — Correlation between RNA-seq and rt-qPCR data to fifteen selected genes. A- Flocculated fermentation (FL); B- Typical fermentation (TF). Samples TF1 and FL1 were used as references in order to obtain expression ratio among TF4 and TF6 and FL4 and FL7 samples, respectively. Expression values were obtained using three techniques replicates and are presented as fold change Log2. [file 12934_2015_196_MOESM5_ESM.png]
