# Supplementary material for: Saccharomyces cerevisiae transcriptional reprograming due to bacterial contamination during industrial scale bioethanol production
Source: Microb Cell Fact. 2015 Jan 30;14:13. doi: 10.1186/s12934-015-0196-6 (PMC4318157; doi:10.1186/s12934-015-0196-6)
Supplement: Additional file 6: Table S5. — Enriched GO terms between fermentations. Gene ontology (GO) terms of the differentially expressed (DE) genes were obtained from SGD (http://www.yeastgenome.org/cgi-bin/GO/goSlimMapper.pl) using the Yeast GO-Slim Process parameters and a cutoff p-value <0.01. Functional GO enrichment terms were obtained using DE genes between fermentations (TF1 vs. FL1; TF6 vs. FL7; TFs vs. FLs). FL: Flocculated fermentation. TF: Typical fermentation. [file 12934_2015_196_MOESM6_ESM.docx]

**Additional file 6.**

| **Sample** | **GO term** | **Genes** |
| --- | --- | --- |
| FLs | Cellular amino acid metabolic process | CIT2, LYS21, SFA1, LYS20, MET32, PRO1, STR3, LYS12, MET28, GSH1, MET3, MET14, MET17, MET2, MET16 |
|  | Biological process unknown | YAR029W, YCL023C, YDR374C, JIP4, RMD6, YER130C, YER137C, YER187W, NQM1, YGR139W, YGR250C, AHT1, IML2, YKL071W, YKL131W, RMA1, YLR311C, YLR312C, STP3, YML131W, YMR103C, YMR254C, APJ1, YNL144C, YNL260C, YNL276C, YNL285W, YPL225W, YPR127W, TDA6 |
| FL1 | Biological process unknown | ACO2, AIM25, AIM46, ALT2, APT2, BSC1, BSC6, DDI2, DDI3, ERR3, GDT1, HSP33, ICS3, IRC18, MHO1, MOH1, NQM1, RDL2, RMA1, RMD6, SET6, SKG6, SPG1, STP4, TDA6, TMA16, UIP5, YBL028C, YBL081W, YBR063C, YCR087C-A, YDL085C-A, YDL157C, YDL177C, YDR056C, YER053C-A, YER187W, YFL052W, YGR001C, YGR079W, YGR127W, YHL012W, YHL026C, YHL044W, YHR045W, YHR202W, YIL024C, YIR035C, YJL077W-B, YJL133C-A, YJR115W, YJR124C, YKL071W, YKR005C, YKR017C, YLR030W, YLR031W, YLR040C, YLR253W, YLR297W, YLR312C, YLR342W-A, YLR352W, YLR413W, YML054C-A, YML131W, YMR206W, YMR262W, YMR310C, YNL095C, YNL144C, YNR075C-A, YOR011W-A, YOR012W, YOR192C-C, YOR378W, YOR389W, YPK3, YPL272C, YPL277C, YPL278C, YPR003C, YPR114W, YPR153W, YPR202W, YTP1 |
|  | Carbohydrate transport | ASK10, HXK2, HXT4, HXT5, HXT8, MAL31, MTH1, SKS1 |
|  | Cellular amino acid metabolic process | ACO1, AGX1, ALT1, ARG5,6, ARG80, CIT1, CIT2, GAP1, GCV2, GDH1, GLN4, GLT1, IDH1, IDH2, IDP1, LYS1, LYS12, LYS14, LYS2, LYS20, LYS21, LYS4, LYS9, MET2, PRO1, SAM1, YFH1 |
|  | Cofactor metabolic process | [ACO1](http://www.yeastgenome.org/cgi-bin/locus.fpl?locus=ACO1), [ACS1](http://www.yeastgenome.org/cgi-bin/locus.fpl?locus=ACS1), [BIO2](http://www.yeastgenome.org/cgi-bin/locus.fpl?locus=BIO2), [BNA2](http://www.yeastgenome.org/cgi-bin/locus.fpl?locus=BNA2), [CIT1](http://www.yeastgenome.org/cgi-bin/locus.fpl?locus=CIT1), [COX10](http://www.yeastgenome.org/cgi-bin/locus.fpl?locus=COX10), [FAA4](http://www.yeastgenome.org/cgi-bin/locus.fpl?locus=FAA4), [FAU1](http://www.yeastgenome.org/cgi-bin/locus.fpl?locus=FAU1), [FDH1](http://www.yeastgenome.org/cgi-bin/locus.fpl?locus=FDH1), [HEM3](http://www.yeastgenome.org/cgi-bin/locus.fpl?locus=HEM3), [IDH1](http://www.yeastgenome.org/cgi-bin/locus.fpl?locus=IDH1), [IDH2](http://www.yeastgenome.org/cgi-bin/locus.fpl?locus=IDH2), [ISA2](http://www.yeastgenome.org/cgi-bin/locus.fpl?locus=ISA2), [ISU2](http://www.yeastgenome.org/cgi-bin/locus.fpl?locus=ISU2), [PYC1](http://www.yeastgenome.org/cgi-bin/locus.fpl?locus=PYC1), [SAM1](http://www.yeastgenome.org/cgi-bin/locus.fpl?locus=SAM1), [YFH1](http://www.yeastgenome.org/cgi-bin/locus.fpl?locus=YFH1), ZWF1 |
|  | Ion transport | CCC1, COT1, CTP1, CTR1, FET5, MEP1, MEP2, MEP3, MID1, QDR2, SUL1, YDL183C, YHM2, YMR279C, ZRT2 |
|  | Response to chemical stimulus | AIF1, ASK10, BOP3, CAT8, CLA4, CNA1, DCR2, ERV1, FAR1, FLR1, GLR1, GRX4, GRX7, HAP3, HXK2, IRE1, MIG2, QDR1, QDR2, RDR1, RGT2, SAN1, SCH9, SKS1, STE3, URM1, YPR036W-A, ZWF1 |
|  | Transmembrane transport | AGP1, CLA4, CTP1, CTR1, ECM22, ERV1, FCY2, FCY21, FEN2, FLR1, FUN26, FUR4, GGC1, MAL31, PET9, QDR1, QDR2, SUL1, SUT1, TIM17, YDR387C, YFL054C, YMR279C, YTA12, ZRT2 |
| FL7 | Biological process unknown | APJ1, BSC2, CMC4, ERR2, ERR3, GRE1, GTT3, HSP31, ICY1, IES6, IML2, JIP4, NQM1, NRP1, RMA1, RRT15, SHE10, STP3, TDA6, YAR028W, YAR029W, YBL036C, YCR024C-B, YDR222W, YER130C, YER137C, YER187W, YGL010W, YGL036W, YGL188C-A, YGR017W, YGR237C, YGR250C, YIR018C-A, YKL068W-A, YKL071W, YLR154C-G, YLR162W, YLR312C, YNL144C, YNL155W, YOR192C-C, YPL014W, YPL225W, YPL229W, YPR127W |
|  | Cellular amino acid metabolic process | ADH1, ALA1, CIT2, DUG3, GSH1, LYS20, MAE1, MET32, PDC1, SAM1, SAM2, SFA1, STR3, UGA2 |
|  | Generation of precursor metabolites and energy | ADH1, COX13, COX5B, ENO1, HOR2, PCL10, PDC1, PFK27, PGK1, PHO85, SGA1, TAR1, TYE7 |
|  | Response to oxidative stress | GSH1, MTL1, MXR1, SIT4, UGA2, YAR1, YJR096W |
|  | Transcription from RNA polymerase II promoter | CCL1, EAF3, FKH2, HMO1, IXR1, MBF1, MET30, MET32, NPL3, NUT2, OAF1, PHO85, RBA50, REB1, ROX3, RPH1, SKS1, SPT15, SPT8, SYC1, YAP5 |
| TF1 | Cellular respiration | COX1, COB, COX2, COX3, TAR1 |
|  | Generation of precursor metabolites and energy | COX1, COB, COX2, COX3, GSY1, PDC5, TAR1 |
|  | Protein folding | SSA1, AHA1, HSP78, SSA4, SSA2, HSP104, CPR6, ERO1, HSC82, STI1, HSP82 |
|  | Transmembrane transport | OLI1, SSA1, HSP78, SSA4, KAR2, SSA2 |
|  | Vitamin metabolic process | THI2,PET18,THI20 |
| TF6 | Biological process unknown | AIM17, AIM19, AIM29, AIM41, BSC1, CNL1, COS1, COS4, COS5, COS6, COS7, COS8, COX26, CPR5, CPR8, CRP1, CTL1, CTR86, DDI3, DSF1, ECL1, FMP33, FMP43, FYV4, HMF1, IRC10, IRC22, IRC24, MIT1, MRH1, MTC7, OM14, PER33, PNS1, PST2, RBD2, RDL1, RDL2, RRT13, RRT8, RTC2, RTN2, SET4, SHH4, TCB1, TCB3, TDA10, TDA4, TDA8, THI74, TIR4, TMA17, TOS1, TOS6, UTR5, YBL029C-A, YBL029W, YBL039W-B, YBR016W, YBR096W, YBR284W, YBR285W, YCL042W, YCL049C, YCP4, YCR015C, YCR099C, YCR100C, YCR101C, YDL027C, YDL218W, YDR034W-B, YDR210W, YEL073C, YET3, YFL051C, YFL052W, YGL194C-A, YGL258W-A, YGR026W, YGR035C, YGR035W-A, YGR066C, YGR117C, YGR127W, YGR201C, YGR204C-A, YHR033W, YHR140W, YHR175W-A, YHR210C, YHR213W, YIL060W, YIL108W, YIL169C, YJL016W, YJL163C, YJR111C, YJR115W, YKL033W-A, YKL091C, YKL096C-B, YKL100C, YKL187C, YLR030W, YLR050C, YLR173W, YLR283W, YLR285C-A, YLR326W, YLR413W, YLR446W, YLR455W, YLR466C-B, YML083C, YMR122W-A, YMR206W, YMR247W-A, YMR262W, YMR317W, YNL011C, YNL058C, YNR014W, YNR061C, YNR065C, YNR071C, YNR073C, YNR075C-A, YOL014W, YOL087C, YOR052C, YOR059C, YOR186W, YOR342C, YOR381W-A, YOR390W, YPL067C, YPL168W, YPL245W, YPL247C, YPL264C, YPL272C, YPR013C, YPR015C, YPR063C, YPR114W, YPR172W, YPS5, YSC83, ZPS1 |
|  | Carbohydrate metabolic process | ACN9, ALG14, ALG6, ARA1, BMH2, CAT8, CAX4, CHS7, CRH1, CTS1, DAK2, DFG10, DOG2, DPM1, EXG1, GAC1, GAL3, GCR1, GDB1, GSY1, GUT1, GUT2, HXK2, ICL1, IMA1, IMA2, KEG1, MAL12, MAL13, MAL32, MAL33, NDE2, OST2, OST3, OST4, PCK1, PCL7, PMI40, PMT4, PSA1, PYC1, PYC2, SIP4, SNF4, SOL3, SOL4, SUC2, SWP1, TDH2, TDH3, TPS3, YIG1, ZWF1 |
|  | Cell wall organization or biogenesis | ECM15, RCR1, TIP1, ROT2, ARP2, PSA1, FMP45, SED1, BMH2 EXG2, PMI40, CRH1, CHS7, SDP1, SIM1, YPS6, CIS3, HSP150, ECM27, CWP1, CWP2, PIR1, ECM4, CCW12, YLR194C, EXG1, ECM19, DFG5, WSC3, HPF1, ECM3, SRL1 |
|  | Cellular amino acid metabolic process | ADI1, ALD2, ALT1, ARG1, ARG4, ARO1, ARO4, ARO8, ASN2, BAT1, CAR2, CAT2, CPA2, DED81, DUG1, ECM38, ECM4, GLT1, HIS1, HIS4, HIS5, HOM3, IDP2, ILV2, ILV5, ILV6, KRS1, LAP3, LEU2, LEU4, LYS1, LYS12, LYS9, MET13, MMF1, MSF1, SAH1, SAM4, SER3, SES1, SPE2, SPE3, THI3, TRP4, TRP5, TYS1, URA2 |
|  | Cofactor metabolic process | PYC2, NDE2, HEM3, SDH4, BNA6, PYC1, RNR4, LSC2, SOL4, COQ6, SOL3, CAB2, GUT2, ACS2, PNP1, HMG1, ZWF1, BIO5, SPE2, LSC1, HEM15, HEM4, ALD4, FDH1, ALD6, SAM4, SPE3 |
|  | Lipid metabolic process | ACB1, ACC1, ADR1, AGP2, ALE1, ALG6, AYR1, CAX4, CDS1, CHO1, CKI1, CRC1, CSH1, DFG10, DPM1, DPP1, ERG20, ERG24, ERG28, FAS1, FEN1, GPI12, GPI18, GPT2, GWT1, HMG1, HMG2, IPT1, IZH4, KAP95, LAP2, MDM31, MGA2, MVD1, NCR1, NTE1, OPI3, ORM1, PHS1, PLB1, PLB2, SAC1, SLC1, STT4, TAZ1, TGL1, TSC13, URA8, YMR210W |
|  | Nucleobase-containing small molecule metabolic process | ADE16, ADE17, ADE8, ALD4, ALD6, APA1, APA2, ARP2, ATP14, ATP7, BNA6, CAB2, FDH1, GUT2, HPT1, IRA1, NDE2, PNP1, PYC1, PYC2, RNR1, RNR2, RNR4, ROY1, SAM4, SEC23, SOL3, SOL4, URA2, URA5, URA8, ZWF1 |
|  | Transmembrane transport | SSA3, BAP2, AGP2, MAL31, ADP1, YCF1, HXT13, AFG3, PIC2, SBH1, YFL054C, SUT1, TOM20, WSC4, CTR2, ATP7, JEN1, PAM17, ATP14, SEC61, TOM40, YMR279C, FET4, PET8, TOM7, SIL1, AUS1, PDR10, CTR1 |
|  | Vitamin metabolic process | BIO5, PET18, PHO3, RIB5, RPI1, SNO2, SNZ3, SPE2, SPE3, THI2, THI20, THI3, THI4 |
| TFs | Amino acid transport | BAP2, TAT1, UGA4, GNP1, CAN1, HNM1, MUP1, GAP1, TAT2, PUT4, SAM3 |
|  | Transmembrane transport | ATP6, OLI1, FLC2, FUI1, SSA3, BAP2, TAT1, PHO89, ADY2, UGA4, ITR1, GNP1, CAN1, FTR1, JEN1, FRE1, CTR3, PDR10, SAM3, SUT2, CTR1 |
|  | Ribosomal large subunit biogenesis | MAK5, REI1 ,SPB1, RSA4, ARX1, NSA2, PRP43, RIX1, IPI3, DBP6, YTM1, NOG1 |
|  | Ion transport | ATP6, OLI1, PHO88, PHO89, ADY2, FTR1, HNM1, FMP43, POR2, PHO90, JEN1, FRE1, CTR3, MEP2, PMA2, CTR1 |
|  | Biological process unknown | YAR062W, YBR016W, GFD2, BSC1, YDL241W, YDR133C, YDR134C, YDR209C, YDR210W, YDR431W, YDR491C, MTC7, UTR5, DSF1, YFL051C, YGL101W, COS12, YGR035C, YGR067C, YGR160W, YGR201C, YGR266W, YGR283C, FSH1, YHR213W, YIL169C, YIL171W, IRC24, YJL027C, YJL107C, PRM10, YJL213W, YKL187C, YKR033C, YLR162W, YLR255C, YMR007W, SEG1, YMR122C, YNL120C, YNL174W, YNR042W, YNR071C, YNR073C, YOL014W, YOL019W, PHM7, ZPS1, TIR4, PNS1, YOR186W, YOR268C, YOR343C, YOR376W, YOR385W, LEE1, YPL136W, RBD2, YPR012W, YPR013C, YPR014C, YPR064W, YPR123C |
|  | Lipid metabolic process | IPT1, INO2, IZH1, CHO1, CAX4, ERG25, CLD1, ERG1, POT1, ERG20, HMG1, ERG13, PLB1 ,MOT3, ERG2, CYB5, ERG24, ARE2, MVD1, ERG10, IDI1 |
|  | Vitamin metabolic process | THI2, PET18, THI13, THI4, THI20, RKI1, SPE3 |
|  | | |
