# Supplementary material for: Saccharomyces cerevisiae transcriptional reprograming due to bacterial contamination during industrial scale bioethanol production
Source: Microb Cell Fact. 2015 Jan 30;14:13. doi: 10.1186/s12934-015-0196-6 (PMC4318157; doi:10.1186/s12934-015-0196-6)
Supplement: Additional file 8: Table S7. — List of genes, PCR products, and primers used in rt-qPCR analysis. [file 12934_2015_196_MOESM8_ESM.docx]

**Additional file 8.**

| **ORF identifier** | **Gene name** | **PCR product size (bp)** |  | **5’-3’ primer sequences** | |
| --- | --- | --- | --- | --- | --- |
|  |  |  |  | **Forward** | **Reverse** |
| YAL005c | *SSA1* | 99 |  | TGCTTACGGTTTGGACAAGA | CCGTCTTCAATGGACAACAA |
| YER062C | *HOR2* | 98 |  | GAGCATCTGGGAATCAGGAG | CCATTCCTGCCCTTCAGATA |
| YER100W | *UBC6* | 99 |  | ATCACAGGACCTGCGGATAC | CATTCTGATAGCCGGTGGTT |
| YFL039C | *ACT1* | 95 |  | CTTTCAACGTTCCAGCCTTC | ATCACCGGAATCCAAAACAA |
| YGL008c | *PMA1* | 98 |  | TGGAGGTCTTCAAAGCATCA | TGCTGTCGAAGGTGCTACTG |
| YGL253W | *HXK2* | 100 |  | AGGTGAAATTTTGCGTTTGG | GTCCATGACGAAAGGCTTGT |
| YHR208W | *BAT1* | 100 |  | TCAAGGAAATTGGCTGGAAC | TCAGCAATCCATTGAGCAAC |
| YIL162W | *SUC2* | 98 |  | ATGACTTGAAGTCCTGGAAG | CTCAGTTGGGACTTCAATCA |
| YNL134c | *-* | 98 |  | ACTTTTCCAGCAGACCCTGA | ACTGGGATGTGGTGGATTTC |
| YNL219C | *ALG9* | 98 |  | GGAATAAGCTGGCATGTGCT | TTGCATGATTCGGTTGATTG |
| YLR248W | *RCK2* | 100 |  | TGCTGTGAAACGTACCGAAG | TTGGGCGCCACTGTAACTAT |
| YLR257w | *-* | 99 |  | GCCCTAAGCAAGATGCAGAA | AACTTCGTCGGTCAGCAAAG |
| YMR186W | *HSC82* | 101 |  | GCCTCCGATGCTTTAGACAA | TCTGGTTTTGGGGTGATTCT |
| YOR374w | *ALD4* | 99 |  | AAATCTTCTGCTGGCTTTGC | ACAAACACCCAAAGGCTGTC |
| YPL239W | *YAR1* | 99 |  | TGGCAAATTAGACGTGGTCA | TTCTCGGCCTCAAAGATAGC |
